# Learnings From Implementation of Technology-Enabled Mental Health Interventions in India: Implementation Report

**DOI:** 10.2196/47504

**Published:** 2024-02-15

**Authors:** Sudha Kallakuri, Sridevi Gara, Mahesh Godi, Sandhya Kanaka Yatirajula, Srilatha Paslawar, Mercian Daniel, David Peiris, Pallab Kumar Maulik

**Affiliations:** 1 George Institute for Global Health New Delhi India; 2 George Institute for Global Health Sydney Australia; 3 Faculty of Medicine University of New South Wales Sydney Australia; 4 Department of Brain Sciences Imperial College London London United Kingdom; 5 Prasanna School of Public Health Manipal Academy of Higher Education Manipal India; 6 George Institute for Global Health London United Kingdom

**Keywords:** mental health, technological interventions, digital health, community intervention, implementation, eHealth, India, Asia, development, health technology

## Abstract

**Background:**

Recent years have witnessed an increase in the use of technology-enabled interventions for delivering mental health care in different settings. Technological solutions have been advocated to increase access to care, especially in primary health care settings in low- and middle-income countries, to facilitate task-sharing given the lack of trained mental health professionals.

**Objective:**

This report describes the experiences and challenges faced during the development and implementation of technology-enabled interventions for mental health among adults and adolescents in rural and urban settings of India.

**Methods:**

A detailed overview of the technological frameworks used in various studies, including the Systematic Medical Appraisal and Referral Treatment (SMART) Mental Health pilot study, SMART Mental Health cluster randomized controlled trial, and Adolescents’ Resilience and Treatment Needs for Mental Health in Indian Slums (ARTEMIS) study, is provided. This includes the mobile apps that were used to collect data and the use of the database to store the data that were collected. Based on the experiences faced, the technological enhancements and adaptations made at the mobile app and database levels are described in detail.

**Implementation (Results):**

Development of descriptive analytics at the database level; enabling offline and online data storage modalities; customizing the Open Medical Record System platform to suit the study requirements; modifying the encryption settings, thereby making the system more secure; and merging different apps for simultaneous data collection were some of the enhancements made across different projects.

**Conclusions:**

Technology-enabled interventions prove to be a useful solution to cater to large populations in low-resource settings. The development of mobile apps is subject to the context and the area where they would be implemented. This paper outlines the need for careful testing using an iterative process that may support future research using similar technology.

**Trial Registration:**

SMART Mental Health trial: Clinical Trial Registry India CTRI/2018/08/015355; https://ctri.nic.in/Clinicaltrials/pmaindet2.php?EncHid=MjMyNTQ=&Enc=&userName=CTRI/2018/08/015355. ARTEMIS trial: Clinical Trial Registry India CTRI/2022/02/040307; https://ctri.nic.in/Clinicaltrials/pmaindet2.php?EncHid=NDcxMTE=&Enc=&userName=CTRI/2022/02/040307

## Introduction

The burden of mental disorders [1] and the treatment gap due to untreated mental disorders in low- and middle-income countries (LMICs) such as India is estimated to range between 75% and 85% [2], with 1 in every 27 individuals being treated for depression [3]. Technological solutions have been advocated to increase access to care, especially in primary health care settings in LMICs, to facilitate task-sharing, given the lack of trained mental health professionals. Research has indicated the effectiveness of employing technologies for addressing complex health concerns among people with mental illnesses. However, the cost-effectiveness of technology-enabled interventions compared to in-person interventions has not yet been established [4].

Technology-enabled service delivery models have increased access to care and facilitated service monitoring, with mobile health (mHealth) being one such strategy. The World Health Organization (WHO) defines mHealth as “a medical and public health practice that is supported by mobile devices, such as mobile phones, patient monitoring devices, and other wireless devices” [5]. mHealth in the form of electronic decision support systems (EDSSs) has been widely adopted by service users and providers for monitoring health status and for diagnosing and managing a range of health conditions, including mental disorders and substance use [6]. mHealth use has increased with increasing penetration of mobile network connectivity [7].

This paper highlights the processes involved in the development and implementation of technology-enabled interventions employed in three projects across rural and urban settings in India among adults and adolescents: the Systematic Medical Appraisal and Referral Treatment (SMART) Mental Health (SMH) Pilot project [8], and two cluster randomized controlled trials (cRCTs), SMH trial [9] and the Adolescents’ Resilience and Treatment Needs for Mental Health in Indian Slums (ARTEMIS) trial [10].

All three projects used EDSSs to facilitate the identification, diagnosis, and management of common mental disorders (CMDs), including depression, anxiety, psychological distress, and increased suicide risk. A task-sharing approach was used where nonphysician health workers known as Accredited Social Health Activists (ASHAs) and primary care doctors worked together to support people at high risk of CMDs [8-10].

This implementation report describes the experiences of using technology in implementing these three mental health projects, following implementation reporting guidelines [11].

## Methods

### Aims and Objectives

This paper highlights the processes involved in the development and implementation of technology-enabled interventions employed in three projects across rural and urban settings among adults and adolescents in India.

### Blueprint Summary

**Figure 1:**
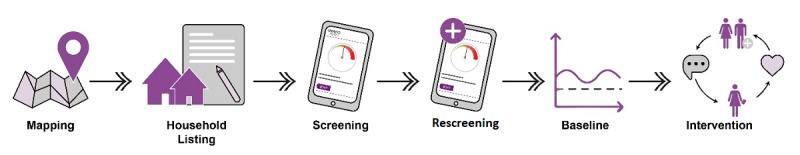
Different phases of the study.

The overall technological framework of the SMH pilot study has two main components: a mobile app and a database. Different mobile apps were developed to collect data at divergent phases of the study (Figure 1). All apps were installed on 7-inch Android tablets for use by ASHAs/community women volunteers (CWVs), or primary health center (PHC) doctors. ASHAs are local women trained from the community with 8th-10th–grade education levels to support the implementation of health programs. While ASHAs work contractually, they are incentivized for their involvement in other projects. CWVs are women who reside in the same community where the study is being done. These CWVs were chosen from the slums and would have similar education level as ASHAs. They were trained on basic knowledge about mental health, along with the stigma and care of individuals with stress, depression, and increased suicide risk.

The three studies underwent a formative phase, testing study tools and mobile apps while gauging user acceptance [12,13]. Iterations were made based on user feedback before the intervention phase. The technical team assessed the app and released a test version for research team testing. Once confirmed, a definitive version was used for data collection.

In the preintervention phase, geographical mapping and demarcation of the village boundaries were performed, followed by house listing to obtain accurate census data. Custom apps were developed for each step, including population screening for identifying individuals at risk of CMDs, which involved data collectors and ASHAs using specific screening tools. After screening, baseline data on various variables were collected before the intervention was implemented (Figure 1).

### Technical Framework Design

The key components of the EDSS included the ASHA app, doctors app, and priority listing app (Table 1). Each ASHA had a finite set of individuals who lived in the geographical location covered by her. The tablets had encrypted, password-protected individual logins unique for every ASHA. Individuals screening positive were referred to primary care doctors for clinical diagnosis and treatment based on predetermined cut-off scores. The doctors used the WHO mhGAP-IG tool (version 1.0) [14] for diagnosing and treating people with CMDs, offering algorithm-based diagnoses and evidence-based treatment recommendations, including comorbidities. Doctors followed these recommendations, entering the type of care provided (pharmacological, psychological, referral, or combinations thereof) into their app. Doctors input the data to generate a traffic light–coded priorities list for ASHAs, indicating the status of screen-positive individuals in their area. Using color coding due to the low education levels of ASHAs, the list included pertinent questions on treatment adherence, social support, and stressors for each color category. The list was dynamic, changing based on doctors’ updates during patient follow-up visits.

**Table 1:** Details of the apps used for the three studies and the target of the intervention.

App		Phase of the study	Users
**SMART MH^a^ (Pilot) and SMART MH trial focused on rural adults**			
	Listing app	Listing (household census data collection)	Data collectors
	Screening app	Household screening for common mental disorders	ASHAs^b^
	Baseline data collection app	Baseline: collected data on different variables and stressors triggering anxiety/depression	Data collectors
	Intervention (ASHA app)	Intervention: for regular follow up of adults at high risk of CMDs^c^ who sought care from the doctor or have yet to seek care	ASHAs
	Intervention (doctor app)	Intervention: diagnosis and treatment for CMDs among adults	Primary care doctors
	3M, 6M, and 12M app	Assessments at 3, 6, and 12 months of the intervention	Data collectors
**ARTEMIS^d^ trial focused on adolescents**			
	Listing app	Listing (household census data collection)	Data collectors
	Screening app	Household screening for common mental disorders	Data collectors
	Baseline data collection app	Baseline: collected data on different variables and stressors triggering anxiety/depression	Data collectors
	Intervention (ASHA app)	Intervention: for regular follow up of adolescents who are at high risk of CMDs who sought care from the doctor or have yet to seek care	ASHAs
	Intervention (doctor app)	Intervention: diagnosis and treatment for CMDs	Primary care doctors
	3M, 6M, and 12 M app	Assessments at 3, 6, and 12 months of the intervention	Data collectors

^a^SMART MH: Systematic Medical Appraisal and Referral Treatment (SMART) Mental Health.

^b^ASHA: Accredited Social Health Activist.

^c^CMD: common mental disorder.

^d^ARTEMIS: Adolescents’ Resilience and Treatment Needs for Mental Health in Indian Slums.

### Identifying an Electronic Medical Record System

**Figure 2:**
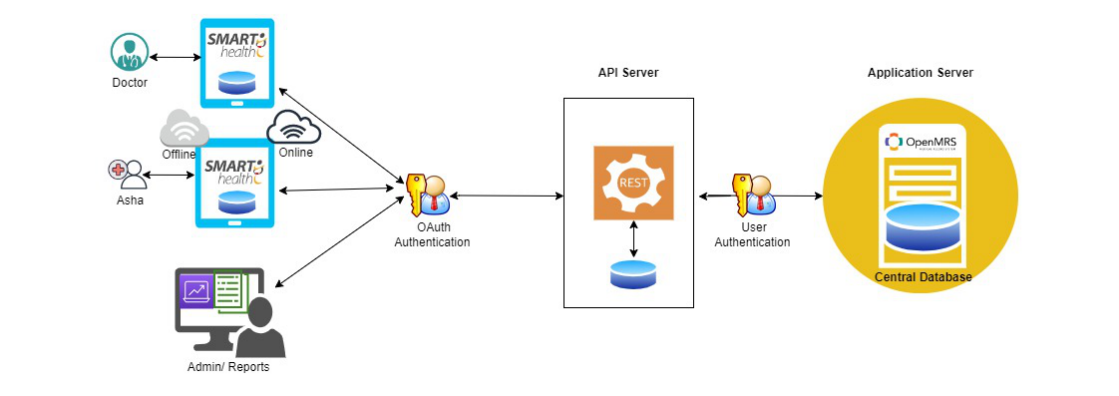
Workflow of data. API: application programming interface; Asha: Accredited Social Health Activist.

All three projects utilized apps based on the Open Medical Record System (OpenMRS) [15], a community-driven open-source software for medical record storage and processing. OpenMRS is robust, scalable for large interventions, and customizable to study workflows and data collection needs. OpenMRS was chosen for these projects as it is freely available. Based on our earlier experience, the functionalities were suitable for our mental health projects [16]. Data collected on tablets underwent authentication and were transferred to the application programming interface (API) server, which were then sent to the application server housing the central OpenMRS database (Figure 2).

### Phases of App Development

**Figure 3:**
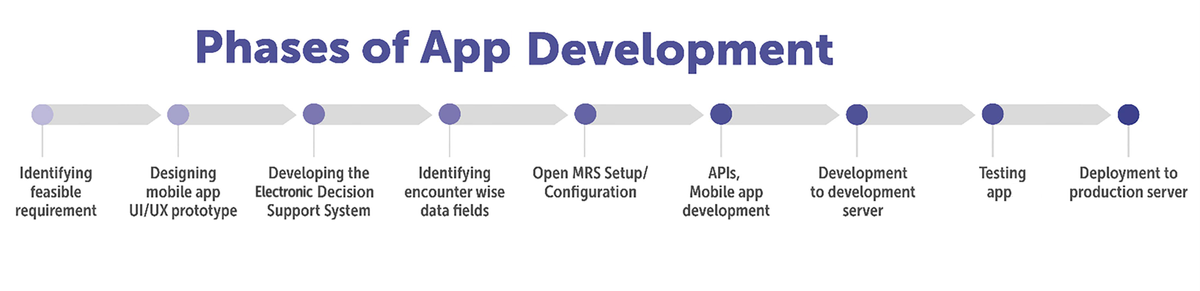
Phases of app development. API: application programming interface; MRS: Medical Record System; UI: user interface; UX: user experience.

The apps went through different phases of development, enhancement, and adaptations across the three projects to suit the specific requirements of each project (Figure 3).

Based on the scope of work (ie, a detailed document or description that outlines the specific tasks, activities, objectives, and deliverables associated with a particular project) received, the technological team assessed requirements and checked the feasibility of incorporating them.

The next step involved the design of the mobile app user interface (UI)/user experience (UX) prototype, which was an interactive mock-up of the mobile app. The prototype contained key UIs, screens, and simulated functions without any working code or final design elements. This provided a better understanding of the real-time UI and UX before production.

Subsequently, the EDSSs were designed according to standard existing diagnosis and management guidelines, which were programmed to develop the most appropriate apps. To identify encounter data fields, individual interactions by ASHAs/doctors were recorded as separate encounters in OpenMRS. Different study phases had distinct data points, necessitating a logical flow of questions. Specific roles were assigned, tailoring the data collection tools to individual responsibilities. For instance, the follow-ups for ASHAs used priority-listing questions, whereas the doctors app incorporated mhGAP tool queries. This ensured targeted and relevant data capture for each study participant.

The next step involved configuring project-specific technical details such as concepts, encounter types, visit frequencies, user roles, and API settings within OpenMRS. Additionally, custom tables were created to facilitate real-time reporting and analytics, ensuring efficient data management and analysis for the project.

**Figure 4:**
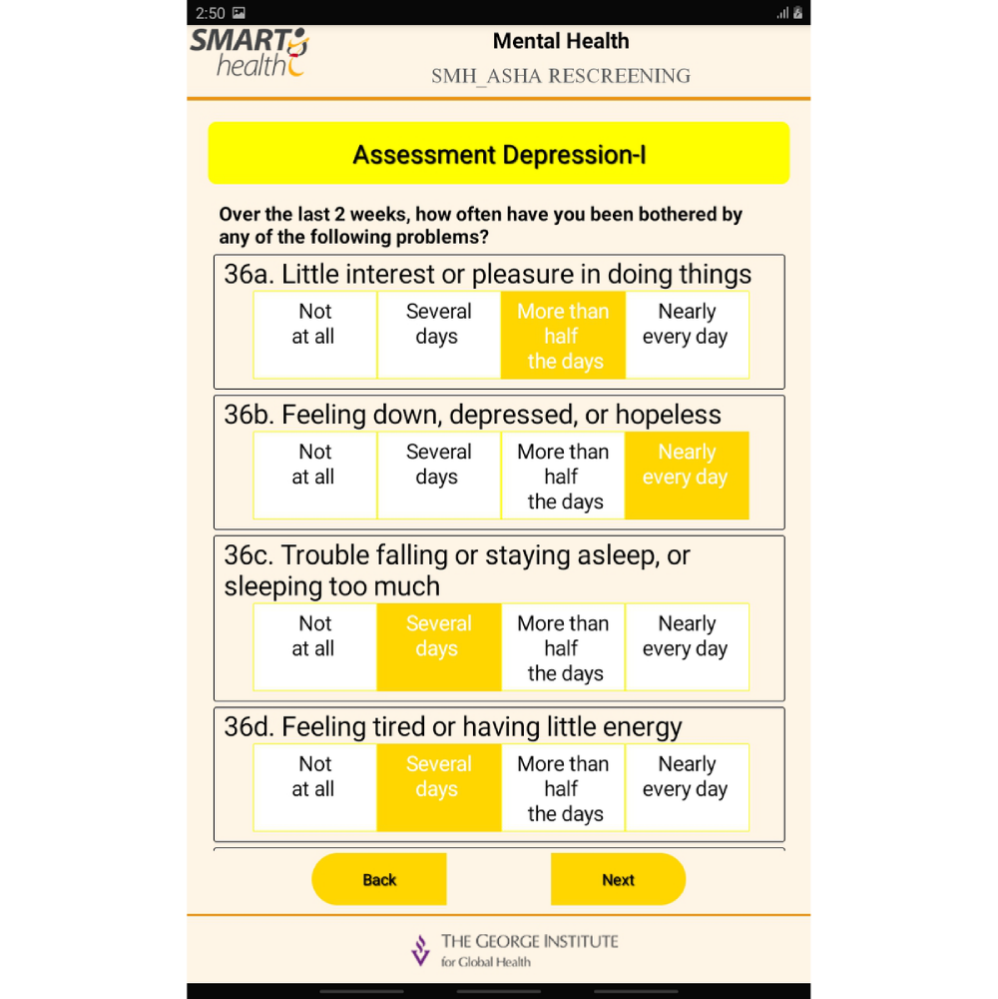
App screenshot 1.

**Figure 5:**
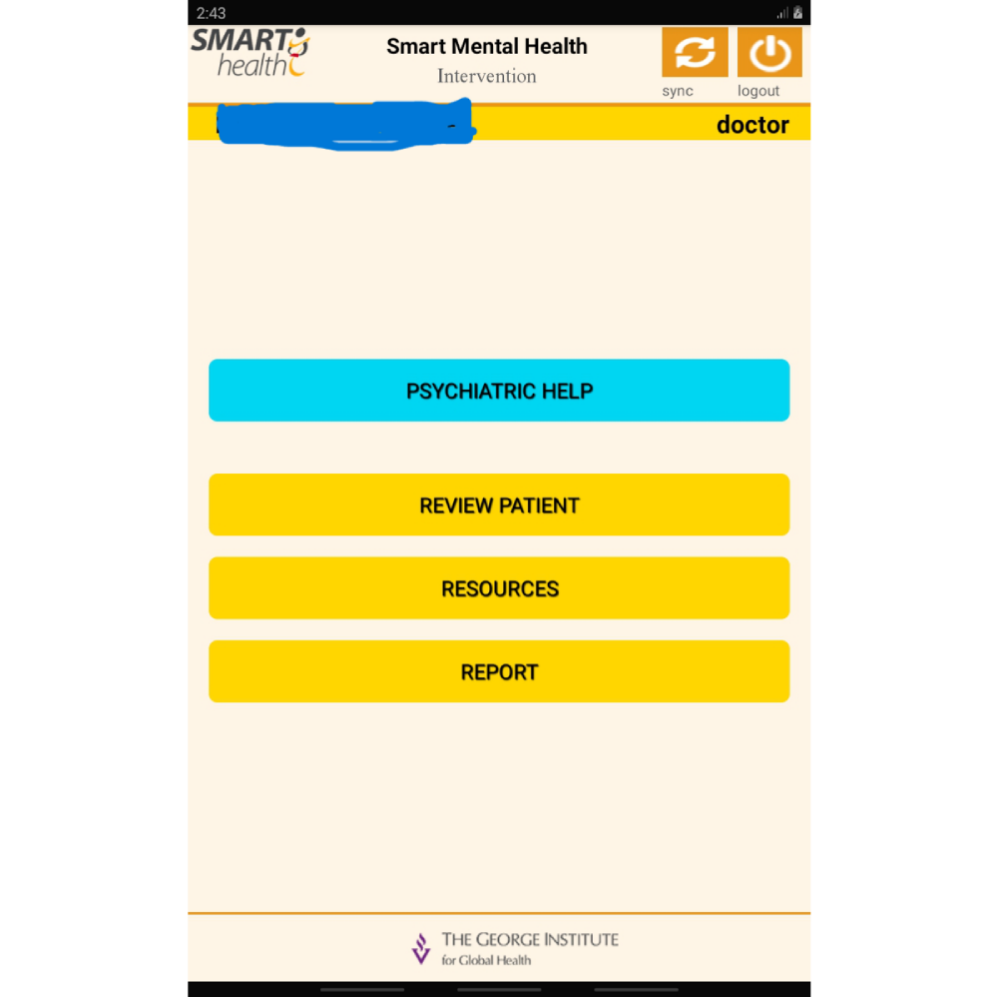
App screenshot 2.

**Figure 6:**
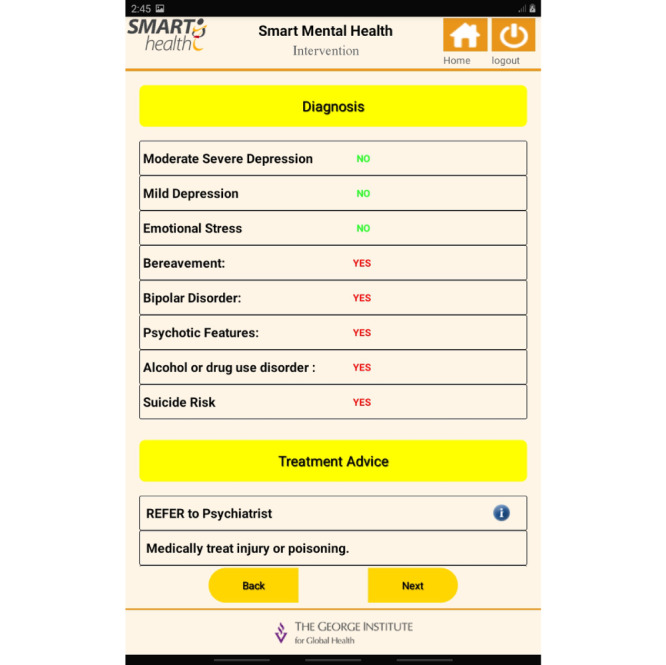
App screenshot 3.

The final step was the development of the mobile app and APIs, which was carried out as a multistage process. The set up followed the sequence of development, test stage, and production environments. The final prototype for the mobile app involved integrating the EDSS into the app. The SMH apps supported online/offline features. Standard security integrations were enabled while developing the mobile app in the local database in the three different environments. In the test environment, the integrated feature was assessed with test data to evaluate the impact of the load of data and the performance of the app. In the stage environment, this phase included an exact replica of a production environment for testing. In the production environment, the software or products were made live for use. Once the development of the app was complete and certified by the quality assurance team, it was deployed for the production environment. Screenshots of the app are provided in Figures 4-6.

Continuous modifications and maintenance of the app were applied across the projects’ lifetimes.

### Target

The SMH Pilot was implemented in 42 villages across rural and tribal areas of Andhra Pradesh [8,17] with the goal of understanding the feasibility and acceptability of using mobile technology and task-sharing approaches to address CMDs. This project covered approximately 50,000 adults and informed the subsequent SMH Trial, which took place in villages across Haryana and Andhra Pradesh, screening 165,000 adults in 133 villages and 44 PHCs. Currently, ARTEMIS is being implemented among 70,000 adolescents (10-19 years old) in 60 urban slum clusters in Vijayawada (Andhra Pradesh) and New Delhi.

### Ethical Considerations

All collected data are securely stored on central servers in Hyderabad, with restricted access limited to the project team. Participants provided written consent and received detailed information about data collection at various time points. The SMART Mental Health pilot study was approved by the Independent Ethics Review Committee of the Centre for Chronic Disease Control (IRB00006330) for studies CCDC_IEC_03_2014 and CCDC_IEC_02_2014 on October 1, 2014; the SMART Mental Health cluster randomized controlled trial was approved by the George Institute Ethics Committee (009/2018) on April 27, 2018; and the ARTEMIS trial was approved by the George Institute Ethics Committee (17/2020) on September 4, 2020. The study tools were approved by The George Institute Ethics Committee, and each participant was assigned a unique identification number at the study’s outset. Data were consistently deidentified before any sharing, and only research staff and the study’s implementation and statistical teams had access to the data, ensuring that confidentiality and ethical standards were maintained throughout the research process.

### Participating Entities

The studies have received funding from various international organizations such as Wellcome Trust/Department of Biotechnology (India Alliance), National Health and Medical Research Council Australia, and the UK Medical Research Council. Importantly, these funders are not involved in data collection or analysis and do not have access to the data. Government agencies, although collaborators, also do not manage or analyze the data. The SMH app is under intellectual property rights of the developer, The George Institute India. Local government consultation occurred for support, but they have no role in data governance.

### Budget Planning

A predefined budget was allocated to the development and implementation of the technological interventions. The main costs incurred included the cost of the server (INR 500,000=US $6862) and the time cost of an Android developer and a technical lead (INR 200,000=US $2868/month for the initial 6 months for development and then a 25% time cost for maintenance). The other costs included the procurement of tablets for data collection.

### Interoperability

The apps used in the three studies followed the Health Level 7 (HL7)/Fast Healthcare Interoperability Resources (FHIR) standards for exchanging patient information between a server and mobile app in JavaScript Object Notation (JSON) format. HL7 has also developed other standards, including the HL7 Clinical Document Architecture. We used FHIR in our apps as it was designed to facilitate interoperability of health care systems, allowing different health care apps and devices to easily exchange and share data. As the FHIR standard is based on modern web technologies such as Representational State Transfer principles, JSON, and Extensible Markup Language, it provides a flexible and scalable approach to health care data exchange, making it easier for developers to build interoperable apps.

### Sustainability

The study was developed and implemented in collaboration with the Andhra Pradesh and Haryana governments. The tool has been previously utilized in two studies with adults while undergoing several phases of enhancements and is currently being used in the ARTEMIS study with adolescents. Poststudy, the tool will be shared with government and other nongovernmental organizations interested in using it.

## Implementation (Results)

### Coverage

The overall coverage of the number of study participants, ASHAs/CWVs, and doctors reached in the three studies is detailed in Table 2.

**Table 2:** Coverage of participants across the three projects.

Project	Study participants reached, n	ASHAs^a^/CWVs^b^ included, n	Doctors included, n
SMART MH^c^ Pilot (2014 to 2019)	50,000 adults	40	14
SMART MH Trial (2018 to 2022)	165,000 adults	175	50
ARTEMIS^d^ (2020-2024)	69,600 adolescents (10-19 years old)	104	27

^a^ASHA: Accredited Social Health Activist.

^b^CWV: community woman volunteer.

^c^SMART MH: Systematic Medical Appraisal and Referral Treatment Mental Health.

^d^ARTEMIS: Adolescents’ Resilience and Treatment Needs for Mental Health in Indian Slums.

### Outcomes and Technical Amendments Made to the Data Repository

OpenMRS indicated the overall number of instances that data were collected for each individual participant at different time points in the study. As OpenMRS has a report generation model, it was difficult to compare different data points for the same person or between participants across the same time point. Hence, an intermediary database was developed in house to facilitate the process of running customized reports, which enabled comparison of data at different time points. This process evolved following considerable testing at the backend to obtain the desired output in terms of data visualization. Some customizations were made to OpenMRS to suit study requirements (Table 3).

**Table 3:** Steps of configurations made to the Open Medical Record System (OpenMRS).

Configuration of OpenMRS modules	Features for the study
Creation of a concept dictionary	Every data point to be used for the study was created as a concept and given a short name
Role management	The roles of each user were fixed and were restricted based on the type of activity they were expected to do; for example, the project manager was only given access to user data management and downloading reports
User management	As per our project flow, the different users were allocated to each role, such as ASHAs^a^, doctors, field staff/data collector, project manager, and administrator
Encounter management	Each entry into the tab for a specific user (ie, ASHA, doctor, data collector) was recorded as an encounter with a unique encounter ID, which helped to differentiate the number of encounters that had taken place for each study participant
Managing encounter types	Based on the different phases of the study, each phase was also considered as a separate encounter, such as the screening, rescreening, ASHA follow-up, and doctor follow-up phases
Manage observations	Each data point was considered as a separate observation
Managing persons	Demographic data for every app user (ASHA, doctor) or participant were stored as person details
Managing patients	In this feature, any additional personal identifiers/demographic details identified could be modified/configured
Cohort management	Specific cohorts were created for every phase of the project, matched to the user. This enabled the users to access data of people who were in their own cohort. This helped them to identify and follow up the individuals easily. This was done both for ASHAs and doctors, with each doctor in a particular PHC^b^ having a defined set of ASHAs, who in turn had a defined set of high-risk individuals
Multilocation data management	This was a custom development made to the system to ensure the data of one location (state) were not merged with data from another location. This was relevant to the SMH^c^ and ARTEMIS^d^ trials, which involved two different geographical locations.

^a^ASHA: Accredited Social Health Activist.

^b^PHC: primary health center.

^c^SMH: Systematic Medical Appraisal and Referral Treatment Mental Health.

^d^ARTEMIS: Adolescents’ Resilience and Treatment Needs for Mental Health in Indian Slums.

In India, internet connectivity varies, particularly in rural regions. To address this, the quality of connectivity was assessed in each study area and hotspots were identified. Offline and online data storage methods were implemented, allowing local storage on tablets in areas with poor connectivity. Data could later be uploaded to the central server once connectivity was restored. Additionally, networks at certain PHCs were improved, increasing the bandwidth to enable ASHAs and doctors to upload data when in proximity to these PHCs.

### Lessons Learned

There were several lessons learned while designing and implementing these interventions, which resulted in several enhancements to the systems for improving UX and achieve the study outcomes. Following the SMH Pilot, issues were identified in the EDSS that needed to be corrected for the SMH and ARTEMIS trials (Table 4). During the project, unforeseen challenges arose due to the COVID-19 pandemic. Face-to-face training for health workers was impossible, leading to the preparation of training materials delivered with the assistance of field staff. Additionally, some tablets used by health workers broke down, necessitating replacements and revealing bugs in the app. The SMH cRCT project faced difficulties because of COVID-19, and different mitigation strategies were adopted to ensure implementation of the different stages of the project. However, due to the rapidly changing situation, those also had to be modified quickly. Considering all the issues encountered earlier, we tried to mitigate all these challenges encountered during the SMH pilot study and cRCT, leading to enhancement of the apps developed for the ARTEMIS project. To have a smooth transition from the test environment to the live environment, the technical team performed additional checks by testing the apps by the field staff and creating data that were uploaded to the server to confirm whether all the fields are being populated correctly. This helped in reducing the errors while data were being captured in live scenarios.

**Table 4:** Enhancements made to the electronic decision support system.

Issues that needed amendments	Solutions for the problems/issues
Daily monitoring of data at the field level and comparison of data across sites, localities, and users was very difficult. Monitoring of clinical data of patients was also difficult	Development of descriptive analytics at the database level while implementing the SMH^a^ trial was done to ease monitoring of data. There were many enhancements made at that level, in terms of representing real-time data from different aspects of the study. This included identification of mental health service use, the burden of different mental health conditions, and comparison of different conditions, among other factors. These analytics could be viewed by comparisons made across regions, gender, and age groups. These were represented through pictorial modes such as graphs and pie charts (see Figure 7 for examples)
Monitoring an individual’s mental health status over time was not possible	Analytics were developed to track the PHQ9^b^ and GAD7^c^ scores of an individual in the different phases of the study. Data captured periodically during monitoring could be viewed as graphs and charts based on the longitudinal data at the backend using analytics.
The performance of ASHAs^d^ could not be tracked well	There were enhancements made to the ASHA app, which tracked the performance of each ASHA and provided data about the numbers of screenings and follow-ups performed, including the time taken for each. Random audio recordings of their interactions were also captured to ensure quality checks.
As the database is encrypted and stored in a password-protected, secure location, it is hard to gain access to data by reverse engineering or decoding	The app is protected with multifactor authentication using a password and lock pin as an enhancement to the existing setup.
User interface and functioning of the app were not clear	Several changes were made to the user interface, including a change of font size, color, and creating different section headers using attractive symbols/pictures, for better user experience
Enabling online training during COVID-19	Some of the training materials were embedded in the mobile apps to enable easier access for trainees using virtual modes during COVID-19.
Real-time monitoring of the activities of field staff was required to ensure increased data quality	Random audio recording of interaction of field staff with study participants or high-risk individuals was enabled. The time taken for each screening was also made available at the database level for these audio recordings. This helped the implementation team to monitor data collection and quality.
Merging of two apps, namely household listing and participant screening, into one app	This merger made it possible for simultaneous data collection for both listing and screening, which saved time for both the participant and field staff and reduced multiple visits to the same household for data collection.

^a^SMH: Systematic Medical Appraisal and Referral Treatment Mental Health.

^b^PHQ9: Patient Health Questionnaire-9.

^c^GAD7: Generalized Anxiety Disorder-7.

^d^ASHA: Accredited Social Health Activist.

**Figure 7:**
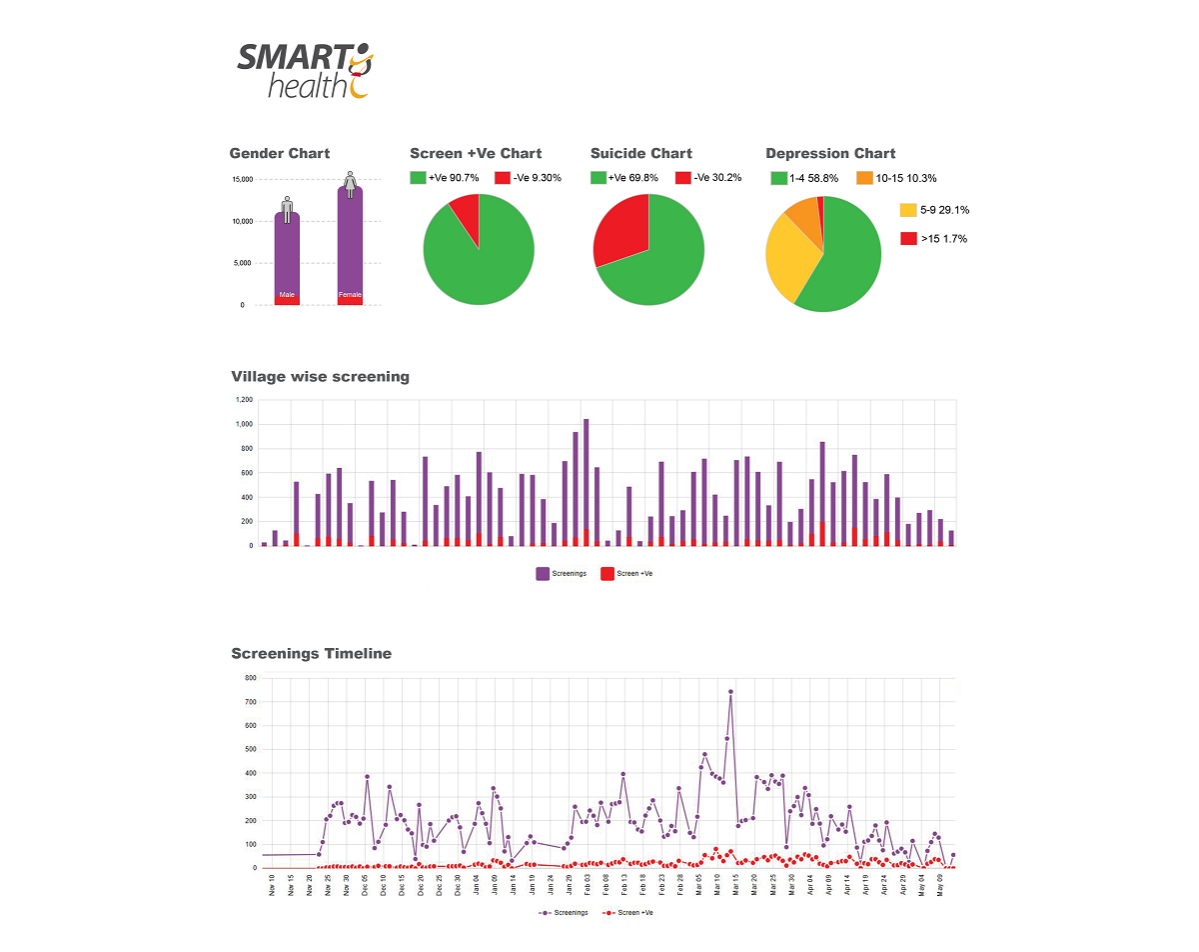
Snapshot of the analytics developed to monitor the progress of the study outcomes. +Ve: positive; -Ve: negative.

## Discussion

### Principal Findings

This paper outlines the experience of employing technology in mental health service delivery across rural and urban India in three projects. We have highlighted the implementation challenges and app adaptations based on user feedback, offering insights valuable for technology-based mental health projects in resource-limited settings. Technology-enabled interventions have shown effectiveness in diagnosing, treating, and following up on various health conditions [18,19]. Most mHealth interventions used in India have been disease-specific and do not involve a health systems approach. One example of a more health system–focused app is the Government to Government web-based monitoring information system that has been set up by the Ministry of Health & Family Welfare, Government of India, to monitor the National Health Mission and other health programs. To increase effectiveness, these innovations should focus on creating new avenues to integrate tools that have encouraging and sustainable outcomes related to access, equity, quality, and responsiveness. The SMH app can be integrated with government systems after specific modifications. The use of electronic medical record systems and telemedicine are examples of some of the interventions implemented and found to be beneficial for health care delivery for large populations, especially in LMICs [16,19,20]. However, there is a need to understand the local context and setting while developing or enhancing any existing app, as some of the original features may not be relevant to the local context, making further adaptations critical.

One way to enhance the functional capabilities of apps such as SMH is to link the app with telemedicine facilities that amplify the ability to connect to remotely located consumers with specialists located in larger cities [13]. For example, machine learning has been applied for suicide prediction, matching patients to appropriate treatment, improving the efficacy of mental health care by clinicians, and monitoring patients for treatment adherence with the help of smartphones and sensors [21].

Another way to leverage technology in mental health is by using artificial intelligence. A recent systematic review recommended the use of artificial intelligence technologies as accurate and effective strategies in the diagnosis and treatment of mental health conditions [22]. Virtual reality technology has proven to be a useful and powerful tool in addiction research [23]. The user interacts with the virtual reality environment, offering an environment close to real life that is dynamic in nature and requires active participation. These environments can be used to develop psychotherapeutic interventions by adding a personal touch, having predictable conditions with additional features such as embodiment, eye tracking, and other biological factors [24].

There is still substantial work to be done in terms of scaling up these interventions and understanding their feasibility and acceptability across different settings and populations. Use of novel strategies such as videogaming can be explored to implement mental health interventions that can be customized to specific populations [25]. Such techniques should be considered in future iterations of the technology platform [26,27].

### Limitations

There were a few limitations in our apps. First, the mobile apps developed were limited to stress, depression, anxiety, and increased suicide risk; however, the principles of including other mental health conditions would be quite similar. Second, although the projects had a system of referring participants requiring specialist care to mental health professionals, it was beyond the scope of the projects to track the care provided by the mental health professionals through our app. This was because our app was developed through primary health system–focused application for use in low-resource settings and was not linked to any central electronic health record system as is possible in more developed health systems with more robust data capture and record-sharing capabilities, such as the National Health Service in the United Kingdom or health systems in Australia. Third, the current apps are compatible on Android platforms and could not be expanded to other operating systems. Finally, the apps developed were specifically created following consultations with local stakeholders; hence, their generalizability across other settings will need to be assessed after adaptation is complete.

### Future Recommendations

Given our experiences, we have compiled a set of suggested recommendations for technology-based interventions in similar settings, which are presented in Textbox 1.

**Recommendations for technology-based interventions.**

- Inclusion of the technical team from the outset when study protocols are being developed.
- All study-related tools and database designing should be finalized in consultation with relevant experts.
- A protocol that details the process of server support in terms of setup/maintenance needs should be developed and followed.
- The server needs to factor in the size of the data set and latest versions of operating systems in reducing any issues faced.
- App user interface/user experience should be designed and assessed for acceptability by targeted populations. The use of reports or data analytics for the study must be discussed and finalized as per study needs.
- Develop systems that can be used across any kind of device, are compatible for software or version upgrades, and are web-based and easily programmable.
- A technical guide with frequently asked questions outlining the various aspects of technology, such as navigation, problem-solving, and reporting of issues, should be developed to facilitate staff training.
- The infrastructure and the architecture of the app should be flexible for making modifications or scaling up the app. The scalability is measured by the number of requests an app can manage and support the app effectively. A decision needs to be taken in terms of adding resources to the computing system for scaling either horizontally (adding more machines to the existing pool) or vertically (adding more power to the existing machines). Both types of scaling are similar as they add computing resources to the infrastructure; however, there are distinct differences between the two in terms of implementation and performance.

### Conclusion

In conclusion, the development of any health-related app is subject to the context and the area where it would be implemented. There is a need for careful testing using iterative processes, allocate human and budgetary resources that are adequate, and integrate apps with larger electronic health record systems that inform health systems.

## Abbreviations

API: application programming interface
ARTEMIS: Adolescents’ Resilience and Treatment Needs for Mental Health in Indian Slums
ASHA: Accredited Social Health Activist
CMD: common mental disorder
CWV: community woman volunteer
cRCT: cluster randomized controlled trial
EDSS: electronic decision support system
FHIR: Fast Healthcare Interoperability Resource
HL7: Health Level 7
JSON: JavaScript Object Notation
LMIC: low- and middle-income country
mHealth: mobile health
OpenMRS: Open Medical Record System
PHC: primary health center
SMH: Systematic Medical Appraisal and Referral Treatment (SMART) Mental Health
UI: user interface
UX: user experience
WHO: World Health Organization
